# Dermoscopic Polymorphism of a Pigmented Eccrine Poroma on the Leg: A Diagnostic Pitfall

**DOI:** 10.7759/cureus.115163

**Published:** 2026-08-25

**Authors:** Sofia Elhaitamy, Meryem Soughi, Salma Kozmane, Layla Tahiri Elousrouti, Fatima Zahra Mernissi

**Affiliations:** 1 Department of Dermatology, Faculty of Medicine, Pharmacy and Dental Medicine, Hassan II University Hospital, Sidi Mohamed Ben Abdellah University, Fez, MAR; 2 Department of Pathology, Hassan II University Hospital, Sidi Mohamed Ben Abdellah University, Fez, MAR

**Keywords:** chalice-form vessels, dermoscopy, frog's egg appearance, melanoma mimic, pigmented eccrine poroma

## Abstract

Pigmented eccrine poroma (PEP) is a rare benign adnexal tumor that can closely mimic melanoma clinically and dermoscopically, particularly when it occurs on non-acral locations. We report the case of a 31-year-old man who presented with a slow-growing, well-circumscribed brown nodule on his left leg that had been evolving for four years. Dermoscopy revealed a polymorphic pattern with chalice-form vessels, perivascular white halos, a frog's egg appearance, and structureless blue and white areas suggestive of melanoma. Histopathological examination confirmed the diagnosis of pigmented eccrine poroma. Complete surgical excision was performed with no recurrence at two-year follow-up. This case highlights the importance of recognizing the characteristic dermoscopic features of PEP to avoid unnecessary aggressive procedures and patient anxiety.

## Introduction

Eccrine poroma (EP) is a benign adnexal neoplasm arising from the intraepidermal portion of the eccrine sweat duct, known as the acrosyringium [1]. It classically presents as a solitary, slow-growing, skin-colored or pink nodule, most commonly located on acral sites such as the palms and soles, reflecting the high density of eccrine glands in these areas [2]. In contrast, the pigmented variant (PEP) represents a distinct and far less common presentation, accounting for approximately 17% of EP cases [3]. Unlike classic EP, PEP occurs more frequently at non-acral locations and is more prevalent in individuals with darker skin phototypes [4], a distribution thought to reflect the presence of melanin within the tumor rather than a distinct clinical entity [1]. This combination of marked pigmentation and an atypical non-acral site makes PEP a notable diagnostic pitfall, as it can closely mimic pigmented malignant lesions. We report a rare case of pigmented eccrine poroma arising on the lower leg of a young man, highlighting its diagnostic pitfalls and distinctive dermoscopic features to increase awareness of this uncommon variant and avoid misdiagnosis as melanoma.

## Case presentation

**Figure 1:**
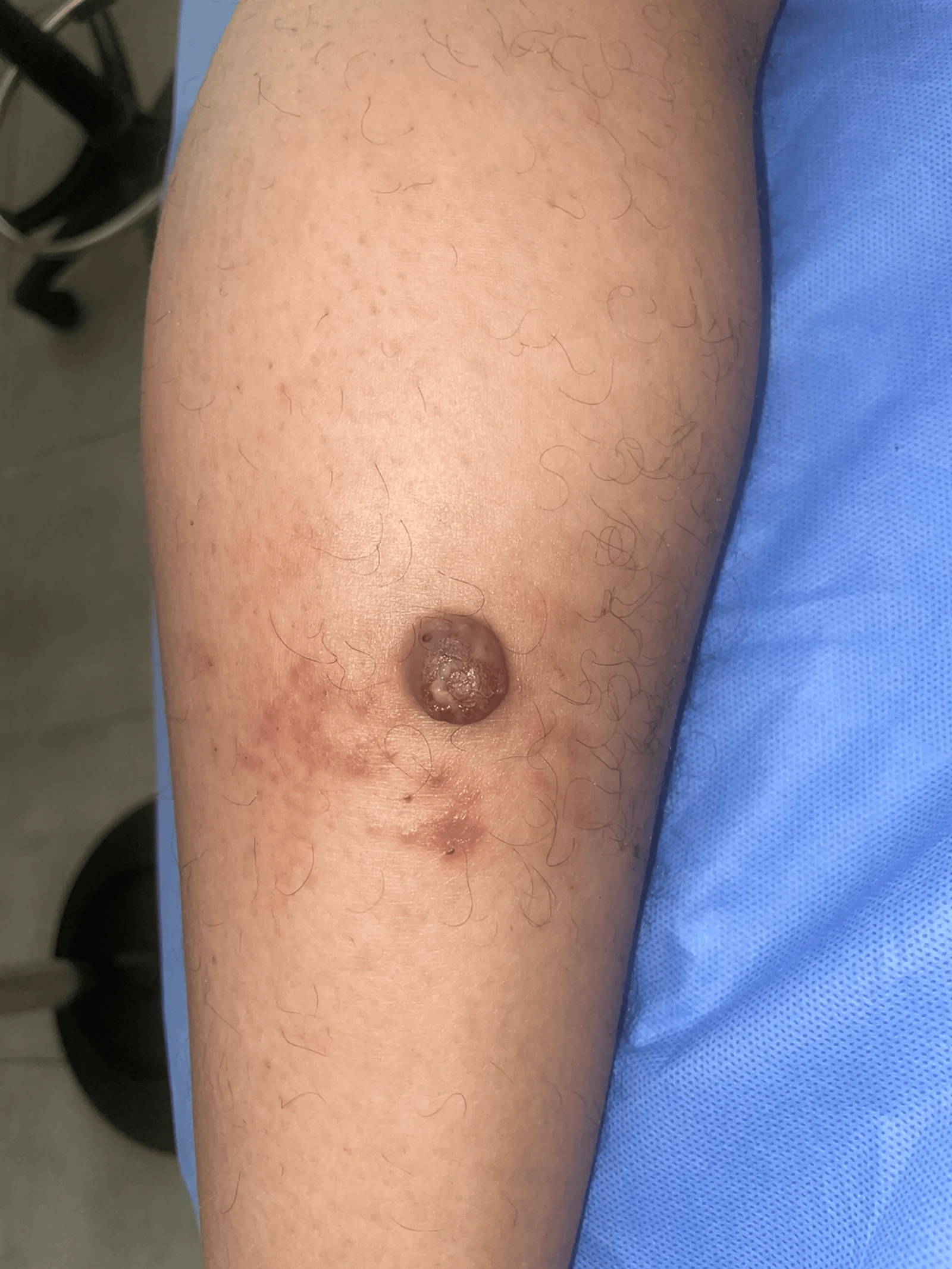
Brownish tumor with an infiltrated base on the left leg

A relatively young 31-year-old man with no medical history or history of trauma presented with a slowly enlarging, firm, well-circumscribed, brown asymptomatic nodule measuring 15 mm in diameter on the left leg, evolving for four years (Figure 1).

**Figure 2:**
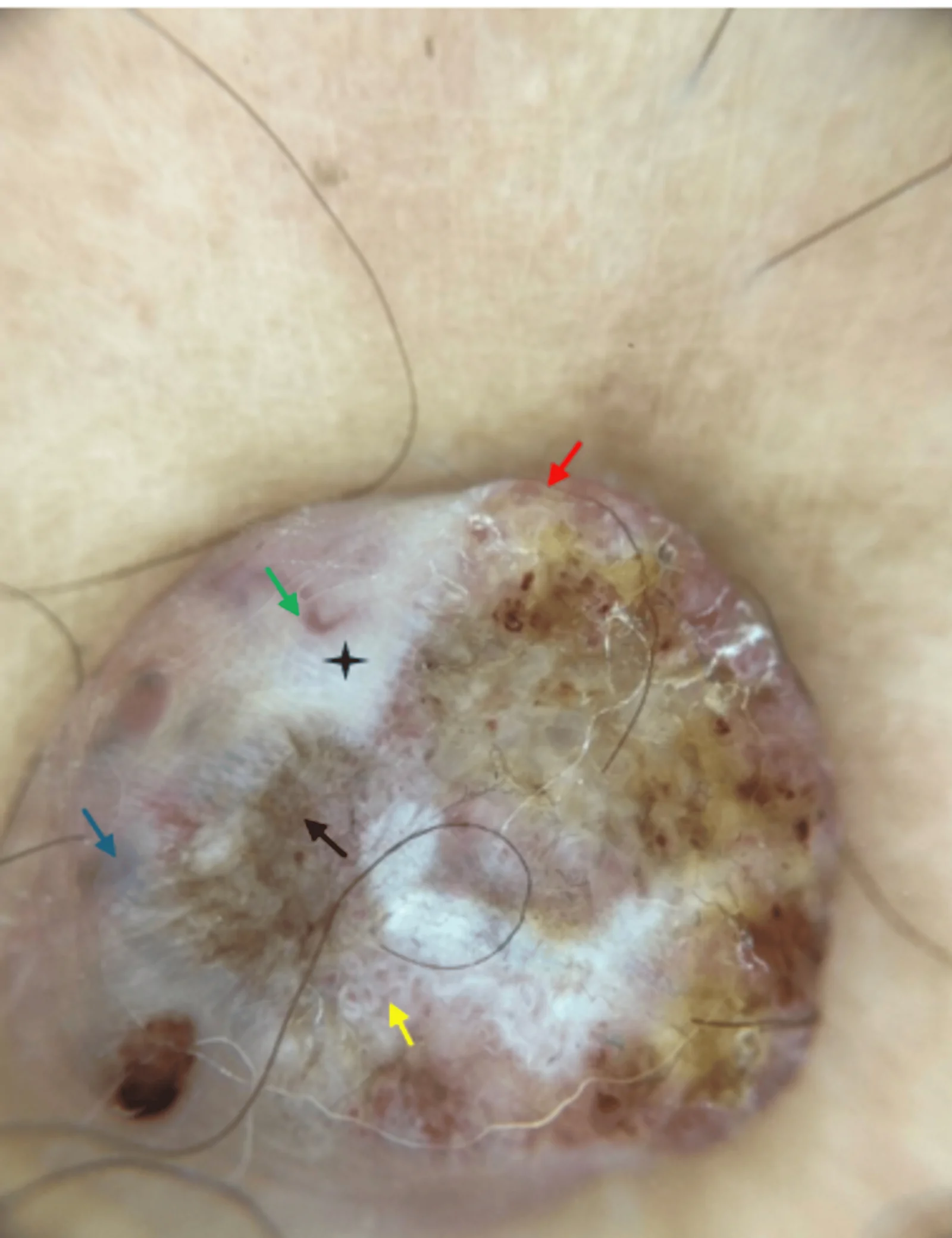
Dermoscopic appearance of the tumor Chalice-form vessels (red arrows), linear vessels (green arrow), and hairpin vessels with perivascular white halo (yellow arrow), structureless white areas (cross), structureless blue areas (blue arrow), and frog egg appearance (black arrows).

Dermoscopy revealed a striking polymorphic pattern characterized by a prominent vascular network composed of chalice-form vessels, linear-irregular vessels, and hairpin vessels, many surrounded by perivascular white halos. Additional features included a characteristic “frog’s egg appearance,” focal hemorrhagic suffusions, and structureless white and brown areas at the center and blue in the periphery (Figure 2).

Systemic examination, including regional lymph node palpation, was unremarkable. Based on the clinical and dermoscopic findings, the main differential diagnoses included melanoma, pigmented basal cell carcinoma (BCC), and squamous cell carcinoma. The lesion was completely excised without a preoperative biopsy, and the diagnosis was established on the excisional specimen. The postoperative course was uneventful, with no complications, and no adjuvant treatment was required.

**Figure 3:**
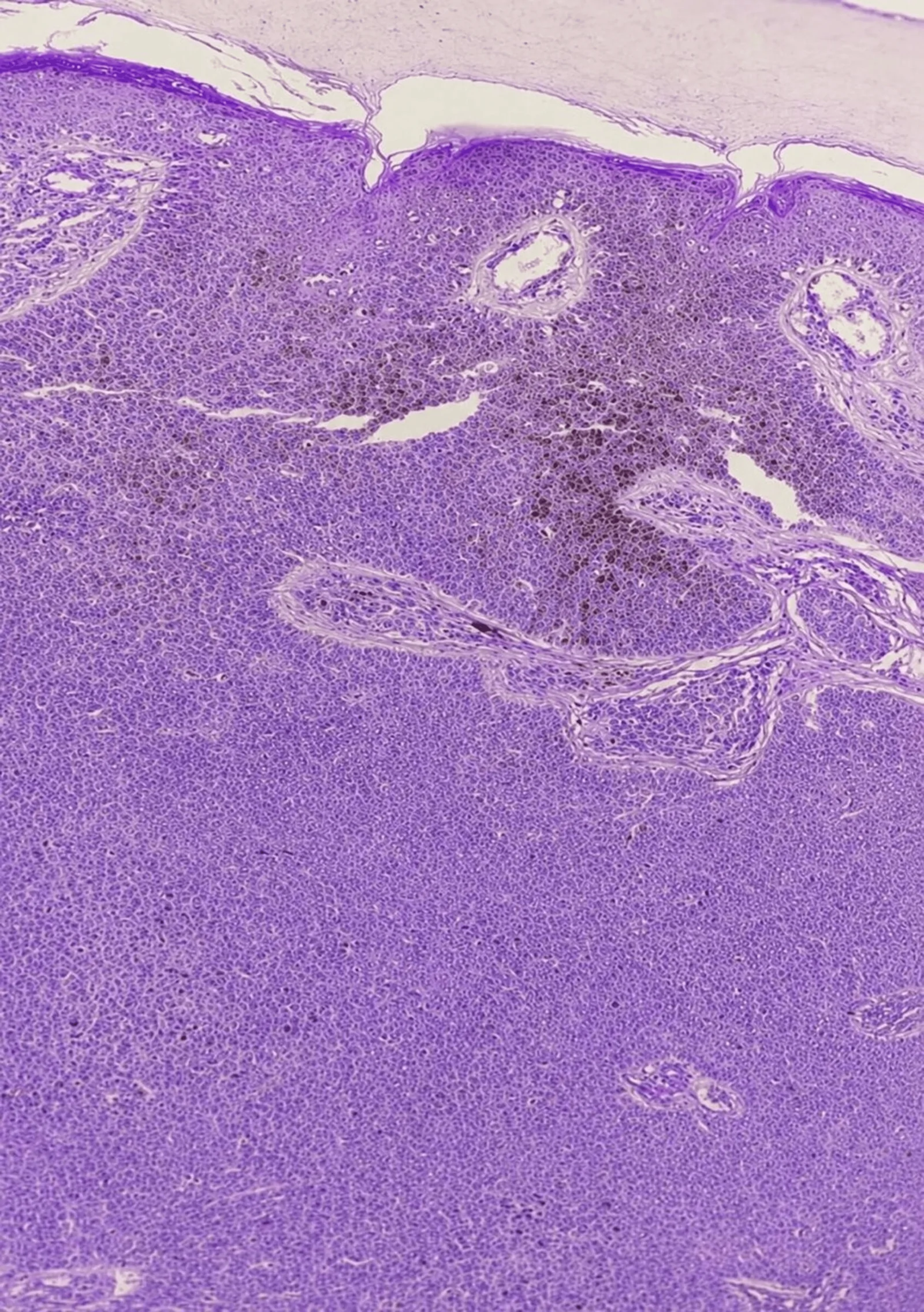
Histopathological findings Broad anastomosing trabeculae of monomorphic basophilic tumor cells connected to the epidermis (H&E stain, magnification x100).

Histopathological examination revealed a tumor connected to the epidermis, composed of broad anastomosing trabeculae of uniform small cuboidal basophilic cells with round nuclei and eosinophilic cytoplasm. Prominent melanin deposition was present within the tumor cells. Although well-defined intratumoral ductal lumina were not clearly identified in the available sections, the characteristic architectural and cytological features were consistent with eccrine poroma. No cytological atypia, mitotic activity, or necrosis was observed, confirming the diagnosis of pigmented eccrine poroma (Figure 3). Immunohistochemical staining was not performed, as the diagnosis was considered sufficiently supported by the characteristic histopathological morphology. No recurrence was observed during two years of follow-up.

## Discussion

EP is an uncommon benign adnexal tumor that predominantly affects adults in the fourth to sixth decades of life [5]. Although its pathogenesis remains incompletely understood, associations with trauma, radiation, or scarring have been reported [6]. Clinically, EP typically presents as a solitary, slowly growing, skin-colored or occasionally pigmented nodule that may ulcerate over time [1]. Histopathologically, it is characterized by a proliferation of poroid epithelial cells with ductal differentiation. In PEP, the presence of melanin within tumor cells and colonization by melanocytes remain poorly understood. Proposed mechanisms include activation of persistent acrosyringeal melanocytes by tumor-derived growth factors, such as endothelin-1, stem cell factor, and nerve growth factor, as well as melanocyte migration from the adjacent epidermis or hair follicles [7].

The pigmented variant of EP poses a significant diagnostic challenge due to its clinical and dermoscopic resemblance to a broad range of pigmented lesions, including melanoma, pigmented BCC, seborrheic keratosis, pigmented melanocytic nevus, and other pigmented adnexal tumors [3,8]. This challenge is even greater when the lesion arises at a non-acral site, such as the lower leg, where poromas are relatively uncommon [9]. Dermoscopy plays a pivotal role as a non-invasive diagnostic tool [4]. Although a polymorphous vascular pattern is the most common dermoscopic feature of EP, distinctive vascular morphologies, including frog's egg, chalice-form, and cherry blossom vessels [10], together with multicolored structureless areas, provide valuable diagnostic clues.

In our case, the differential diagnosis included melanoma and pigmented BCC, both of which can mimic PEP clinically and dermoscopically [3,9]. The polymorphic vascular pattern and structureless blue and white areas initially raised concern for melanoma, particularly given the pigmentation and the non-acral location, where poromas are rarely encountered [9]. However, several dermoscopic features favored the diagnosis of PEP over its malignant mimickers. The presence of chalice-form vessels, hairpin vessels with perivascular white halos, and a characteristic frog's egg appearance provided important diagnostic clues suggestive of eccrine poroma [4,10]. Notably, the blue structureless areas in our case mimicked a blue-white veil, and the white structureless areas resembled regression structures, which further complicated the differentiation from melanoma. Nevertheless, the persistence of the characteristic vascular features of EP, particularly chalice-form vessels and perivascular white halos, was crucial in favoring the diagnosis despite the marked pigmentation.

Differentiating PEP from pigmented BCC can be challenging because of the overlap in dermoscopic features between adnexal tumors and BCC, particularly in skin of color [11]. In this setting, the pearly, translucent quality of BCC contrasts with the more skin-colored or brown surface typically seen in adnexal tumors, while blue-gray ovoid nests and multiple blue-gray or black dots are comparatively more specific to BCC. In contrast, pigmentation in adnexal tumors such as poroma may present as brown structureless areas or green-white ovoid nests [11]. In our case, the absence of arborizing telangiectasias and blue-gray ovoid nests, together with the persistence of chalice-form vessels and perivascular white halos, was decisive in favoring PEP over pigmented BCC.

PEP must also be distinguished from its own malignant counterpart, eccrine porocarcinoma, which can arise de novo or from a long-standing poroma. Dermoscopically, invasive porocarcinoma tends to show a focal presence of whitish-pink structureless areas surrounded by pinkish-white halos, whereas in benign poroma, these same structures are diffusely distributed, giving rise to the classic "frog's egg" pattern; in situ or early porocarcinoma can even display a transitional pattern between the two [12].

Histopathological examination ultimately confirmed the diagnosis of pigmented eccrine poroma by demonstrating broad anastomosing trabeculae of poroid cells connected to the epidermis, with prominent melanin deposition and no cytological atypia, mitotic activity, or necrosis. The absence of malignant melanocytic or basaloid features definitively excluded melanoma and pigmented BCC. This case illustrates the importance of recognizing the characteristic dermoscopic features of PEP and correlating them with histopathological findings to avoid misdiagnosis and unnecessary aggressive management.

Although EP is a benign tumor, it carries a small but significant risk of malignant transformation to eccrine porocarcinoma, reported in up to 18% of cases over an average of 8.5 years [3]. Porocarcinoma is associated with lymph node metastasis in 20% of cases and a mortality rate of up to 67% [13]. Complete surgical excision with narrow margins remains the treatment of choice to achieve clear margins and minimize local recurrence [4]. Regular clinical and dermoscopic follow-up is essential, particularly for lesions showing rapid growth, pain, bleeding, ulceration, or pruritus, which should prompt repeat biopsy to exclude malignant transformation [1].

## Conclusions

In conclusion, this case highlights that PEP should be considered in the differential diagnosis of slowly evolving pigmented nodules, even when located on non-acral sites. Characteristic dermoscopic features, particularly the combination of chalice-form vessels and a frog's egg appearance, can raise suspicion for this diagnosis and provide valuable diagnostic clues. However, given the substantial clinical and dermoscopic overlap with melanoma and other pigmented tumors, histopathological examination is essential for definitive diagnosis. Complete surgical excision is the treatment of choice, offering an excellent prognosis while minimizing the risk of malignant transformation to porocarcinoma. This case further underscores the value of dermoscopy as a complementary non-invasive diagnostic tool in atypical presentations and emphasizes the need for increased awareness of this uncommon variant among dermatologists. Larger prospective studies are needed to further characterize the dermoscopic spectrum of pigmented eccrine poroma, particularly at non-acral sites, and to validate the diagnostic value of chalice-form vessels and the frog's egg appearance in distinguishing it from melanoma and pigmented BCC.
